# 18F-FDG-PET/CT Scan for Detection of Large Vessel Involvement in Giant Cell Arteritis: Arteser Spanish Registry

**DOI:** 10.3390/jcm13206215

**Published:** 2024-10-18

**Authors:** Paula Estrada, Marta Domínguez-Álvaro, Rafael B. Melero-González, Eugenio de Miguel, Maite Silva-Díaz, Jesús A. Valero, Ismael González, Julio Sánchez-Martín, Javier Narváez, Eva Galíndez, Javier Mendizábal, Carlota L. Iñiguez-Ubiaga, Luis Rodríguez-Rodríguez, Javier Loricera, Alejandro Muñoz, Patricia Moya-Alvarado, Patricia Moran-Álvarez, Vanessa A. Navarro-Ángeles, Carlos Galisteo, Santos Castañeda, Ricardo Blanco, on behalf of ARTESER Project Collaborative Group

**Affiliations:** 1 Rheumatology Department, Complex Hospitalari Universitari Moisès Broggi, Universidad de Barcelona (UB), 08970 Barcelona, Spain; paulavestradaa@gmail.com (P.E.); vanavarroa@csi.cat (V.A.N.-Á.); 2 Research Unit, Sociedad Española de Reumatología, 28001 Madrid, Spain; marta.dominguez@ser.es; 3 Rheumatology Department, Complejo Hospitalario Universitario de Vigo, 36312 Vigo, Spain; rafabmg@yahoo.es; 4 Rheumatology Department, Hospital Universitario La Paz, 28046 Madrid, Spain; eugenio.demiguel@gmail.com; 5 Rheumatology Department, Complejo Hospitalario Universitario de A Coruña, 15006 A Coruña, Spain; maitesd@hotmail.com; 6 Rheumatology Department, Hospital Universitario Donosti, 20014 Donosti, Spain; jesval1204@gmail.com; 7 Rheumatology Department, Hospital Universitario de León, 24008 León, Spain; ismamh10@gmail.com; 8 Rheumatology Department, Hospital Universitario 12 de Octubre, 28041 Madrid, Spain; jsm132@hotmail.com; 9 Rheumatology Department, Hospital Universitari Bellvitge, Hospitalet de Llobregat, 08907 Barcelona, Spain; fjnarvaez@bellvitgehospital.cat; 10 Rheumatology Department, Hospital Universitario de Basurto, 48013 Bilbao, Spain; evagalindez@gmail.com; 11 Rheumatology Department, Complejo Hospitalario de Navarra, 31008 Pamplona, Spain; mendizabaljavi@hotmail.com; 12 Rheumatology Department, Hospital Universitario Lucus Augusti, 27003 Lugo, Spain; carlota.laura.iniguez.ubiaga@sergas.es; 13 Rheumatology Department, Hospital Clínico San Carlos, 28040 Madrid, Spain; lrrodriguez@salud.madrid.org; 14 Rheumatology Department, Hospital Universitario Marqués de Valdecilla, IDIVAL Immunopathology Group, 39008 Santander, Spain; jmedtor@hotmail.com; 15 Rheumatology Department, Hospital Universitario Virgen del Rocío, 41013 Sevilla, Spain; alejandrogaleno@hotmail.com; 16 Rheumatology Department, Hospital Santa Creu i Sant Pau, 08025 Barcelona, Spain; pmoyaa@santpau.cat; 17 Rheumatology Department, Hospital Universitario Ramón y Cajal, 28034 Madrid, Spain; pmoranalv@hotmail.com; 18 Rheumatology Department, Hospital Universitario Parc Taulí, 08208 Sabadell, Spain; cgalisteo@tauli.cat; 19 Rheumatology Department, Hospital Universitario de La Princesa, IIS–Princesa, 28006 Madrid, Spain; scastas@gmail.com

**Keywords:** 18F-FDG-PET/CT scan, large vessel vasculitis, aortitis, giant cell arteritis, imaging, nuclear medicine

## Abstract

**Background/Objectives:** Imaging studies have transformed the diagnosis of large vessel vasculitis (LVV) involvement in giant cell arteritis (GCA). A positron emission tomography/computed tomography (PET/CT) scan with 18-fluorodeoxyglucose (18F-FDG) has emerged as a valuable tool for assessing LVV. We aimed to determine the utility of an 18F-FDG-PET/CT scan in detecting LVV in GCA in the ARTESER registry. **Methods**: The ARTESER study is a large multicenter, retrospective, longitudinal, and observational study, promoted by the Spanish Society of Rheumatology. It included patients newly diagnosed with GCA across 26 tertiary hospitals from 1 June 2013 to 29 March 2019. Patients with a diagnosis of incidental GCA were included if they fulfilled specific criteria, including the ACR 1990 criteria, positive imaging examinations, or the expert clinical opinion of investigators. Differences between patients with positive and negative 18F-FDG-PET/CT scan results were analyzed using a bivariate model. A regression model assessed associations in patients with a positive scan, and the predictive capacity of the cumulative dose of glucocorticoids (GC) on PET scan outcomes was evaluated using ROC curve analysis. **Results**: Out of 1675 GCA patients included in the registry, 377 met the inclusion criteria of having an 18F-FDG-PET/CT scan. The majority were diagnosed with a cranial GCA phenotype, and 65% had LVV. The thoracic aorta was the most frequently affected. Cardiovascular disease, diabetes, and older age had a negative association with a positive scan outcome. The OR for having a positive 18F-FDG-PET/CTC scan was lower as the number of days increased. Depending on the cumulative dosage of the GC, the 18F-FDG-PET/CT scan showed an AUC of 0.74, with a Youden index > 60 mg/day. **Conclusions**: Younger patients showed a higher probability of presenting LVV as detected by the 18F-FDG-PET/CT scan. The timing of the examination and the cumulative dosage of the GC influenced the likelihood of a positive result, with earlier tests being more likely to detect inflammation.

## 1. Introduction

Giant cell arteritis (GCA) is the most common primary vasculitis in adults from developed countries in northern latitudes [1]. This condition targets medium- and large-sized arteries. It is predominantly diagnosed in women, and the average age at diagnosis ranges between 70 and 80 years [2,3,4].

The clinical presentation of GCA encompasses a broader spectrum than initially thought. The classic cranial phenotype (cGCA), associated with the involvement of carotid artery branches, is characterized by symptoms such as headache, jaw claudication, and visual disturbances. In contrast, the extracranial phenotype of GCA (ecGCA) is often characterized by polymyalgia rheumatica and systemic inflammatory symptoms, such as fever and weight loss, resulting from the involvement of the aorta and/or its main branches, which may also lead to limb claudication. Sometimes, these two phenotypes can coexist [5]. Aortitis, which can be seen in both the cranial and extracranial form can lead to severe complications, including aortic aneurysms that pose a significant risk of rupture or dissection, leading to high mortality [6].

Recognizing the involvement of large vessel vasculitis (LVV) significantly impacts the evaluation and follow-up of patients with GCA. The sensitivity of the physical examination for detecting LVV ranges from 14% to 50%, underscoring the critical role of imaging modalities in the accurate diagnosis and management of this condition [7]. Patients with aortic involvement in GCA experience a higher frequency of relapses, an increased risk of cardiovascular complications, and they are exposed to higher cumulative doses of glucocorticoids, contributing to a more challenging disease course and management [8].

Temporal artery biopsy (TAB) has been the technique of choice to confirm a diagnostic suspicion of cGCA. The 2022 ACR/EULAR criteria for the classification of GCA represent a significant advancement in the standardization and accuracy of diagnostic protocols, particularly in leveraging imaging techniques [9]. These updated criteria are designed to improve the diagnostic specificity and sensitivity, reflecting contemporary advances in medical imaging and understanding of the disease pathology. This is crucial, as early diagnosis and treatment are pivotal in preventing the more severe complications associated with GCA, such as vision loss and stroke, thereby improving overall patient outcomes [5,10].

The integration of these novel imaging methods, especially the 18F-FDG-PET/CT scan, has significantly enhanced the diagnostic accuracy for the extracranial phenotype of GCA, particularly in detecting the involvement of the aorta and its major branches [11].

The 2023 update from the European League Against Rheumatism (EULAR) has further emphasized the importance of using imaging methods in the diagnosis of LVV [10,12]. These recommendations have catalyzed a broader adoption of such techniques in routine clinical practice, reflecting a shift towards more precise and early diagnosis [5]. Consequently, this shift not only aids in the effective stratification of treatment options but also significantly contributes to the refinement of patient management strategies.

18F-FDG-PET/CT is a functional imaging technique widely used in oncology that has also proven valuable in the assessment of inflammatory diseases. This technique detects increased glucose uptake, a marker of heightened glycolytic activity in inflammatory cells within the affected arterial walls, synovium, or bursae. As a result, FDG-PET/CT can identify systemic LVV in patients with GCA, as well as inflammation in peri-articular and extra-articular synovial structures in cases of polymyalgia rheumatica (PMR) [13,14]. Nevertheless, it is expensive, results in radiation exposure, and is not always available [14].

Considering these factors, we aimed to evaluate the utility of the 18F-FDG-PET/CT scan in detecting LVV among patients with GCA in one of the largest clinical registries of patients with GCA to date.

## 2. Materials and Methods

### 2.1. Study Design

ARTESER (National Registry of Giant Cell ARTEritis of the Sociedad Española de Reumatología (SER) [Spanish Giant Cell Arteritis Registry]) is a large Spanish multicenter, retrospective, longitudinal, and observational study, promoted by the Spanish Society of Rheumatology, including patients with a new diagnosis of GCA from 1 June 2013 to 29 March 2019. Data were obtained through a review of the electronic medical records of patients diagnosed with GCA in 26 hospitals of the Spanish National Health System.

### 2.2. Study Population

The ARTESER registry included all patients with a diagnosis of incidental GCA on the specified dates who met (1) at least 3 out of 5 ACR 1990 criteria [15]; or (2) positivity in one of the following imaging examinations: temporal artery ultrasound, imaging test for large vessels like 18F-FDG-PET/CT scan, computed tomography angiography (CT-A), magnetic resonance imaging angiography (MRI-A), or subclavian or axillary ultrasound with compatible clinical manifestations; or (3) an investigator’s expert clinical opinion. During the recruitment phase, participating hospitals generated a list of patients diagnosed with GCA from their databases. This list encompassed patients across various departments including rheumatology, internal medicine, ophthalmology, and neurology. Access to each patient’s electronic health record was secured to facilitate data collection aligned with the study’s goals. Local investigators were tasked with rigorously reviewing both in-hospital and outpatient coded diagnoses to ensure the inclusion of all newly diagnosed cases of GCA. Subsequently, a subset of patients who underwent an 18F-FDG-PET/CT scan was identified for further analysis. The primary outcome of this study was the detection of vasculitis through the utilization of the 18F-FDG-PET/CT scan imaging modality.

### 2.3. Variables

For this study, the variables recorded from the ARTESER registry were as follows: (a) demographics (age at diagnosis, sex); (b) cardiovascular risk factors (smoking, obesity, diabetes mellitus [DM], hypertension [HT], dyslipidemia [DLP], previous cardiovascular disease [CVD]); (c) laboratory test results such as acute phase reactants (erythrocyte sedimentation rate [ESR] and C-reactive protein [CRP]), hemoglobin, platelet count; (d) cumulative dose of GC before the examination; and (e) the time between the symptoms’ onset and the performance of the imaging test. To calculate the total accumulated GC dose before the 18F-FDG-PET/CT scan, a calculator, designed specifically for this study (Corti-SER) based on detailed periods with the precise dose of GC administered, was used [16].

The clinical phenotypes were predefined as cGCA or ecGCA, depending on the predominant symptom at diagnosis, as collected in the basal visit from the registry: (a) cGCA, when headache, occipital pain, and/or jaw claudication were present and (b) ecGCA, when there were predominantly extracranial manifestations such as upper and/or lower extremities claudication, PMR following the ACR/EULAR 2012 criteria [17], and constitutional symptoms such as asthenia, anorexia, weight loss, and low-grade fever/fever, with none of the cranial manifestations mentioned above.

### 2.4. Statistical Analysis

The results are presented as a bivariate model to analyze the differences between patients with positive and negative results. Continuous variables were presented as the mean with their standard deviation (SD) or as the median and interquartile range, as appropriate, while the categorical variables were represented using absolute frequencies and their respective percentages.

A regression model, adjusted by HT, DM, DLP, and CVD, approaching the association between variables (clinical phenotype, previous accumulated GC dose, and days elapsed before the 18F-FDG-PET/CT scan) in those with a positive test, was used, where an odds ratio (OR) with a 95% confidence interval (CI) was calculated. A receiver operating characteristic (ROC) curve analysis was used to assess the predictive capacity of the cumulative dose of GC on a PET scan and the area under the curve (AUC). Youden’s index was used to determine the best cutoff point.

### 2.5. Ethical Aspects

The study protocol was approved by the Ethics Committee of the referral center at Santander (Spain) and by the Local Ethics Committees of the participating hospitals as required by the Spanish legislation. This study was developed following Good Clinical Practice standards and subject to the ethical principles outlined in the Helsinki Declaration.

## 3. Results

### 3.1. General Features of the Studied Population

From the ARTESER registry, encompassing 1675 patients diagnosed with giant cell arteritis (GCA), 377 met the inclusion criteria having undergone an 18F-FDG-PET/CT scan during their diagnostic process. Of these, 322 patients, or 85%, satisfied the ACR/EULAR 2022 classification criteria for GCA. As detailed in Table 1, the bivariate analysis revealed that 66% of the patients were diagnosed with cGCA, while 29% exhibited the ecGCA phenotype. Additionally, 19 patients, which represented 5% of the sample, could not be classified into either phenotype.

Sixty-five percent of patients in our registry had LVV, as detected by the 18F-FDG-PET/CT scan (n = 245). In those patients with a positive 18F-FDG-PET/CT scan, the vascular territory most frequently affected was the thoracic aorta (85.7%), followed by the supra-aortic vessels (78.8%), and the abdominal aorta (57.6%). CVD, defined as a coronary event or stroke, was more frequent in patients with a negative 18F-FDG-PET/CT scan (Table 1).

### 3.2. Multivariate Analysis

The regression model for patients with GCA for whom an 18F-FDG-PET/TC scan was performed did not find variables associated with a positive 18F-FDG-PET/CT scan (Table 2). CVD, DM, and older age had a negative association with a positive 18F-PET-CT scan (OR 0.439 [0.211–0.914], 0.482 [0.238–0.978], and 0.949 [0.914–0.986], respectively). In addition, the OR for having a positive 18F-FDG-PET/CTC scan was lower as the number of days increased.

**Figure 1:**
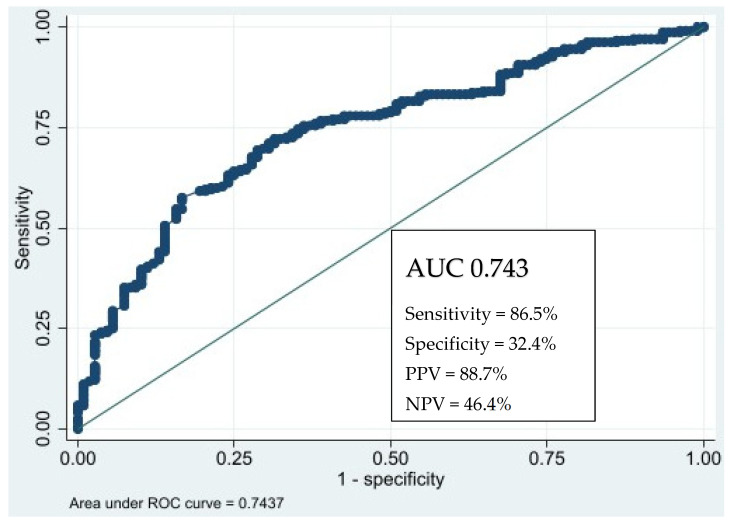
Results of the area under curve in the ROC analysis. ROC: receiver operating characteristics. PPV: positive predictive value. NPV: negative predictive value.

In this study, we examined the influence of the cumulative GC dosage on the 18F-FDG-PET/CT scan outcomes. The analysis revealed an AUC of 0.74 (Figure 1), indicating a moderate predictive accuracy. The optimal cut-off point, as determined by the Youden index, was established at a cumulative GC dosage ≥ 60 mg/day. At this threshold, the 18F-FDG-PET/CT scan exhibited the highest sensitivity and specificity, with values of 86.53% and 32.41%, respectively.

Further analysis showed that a cumulative dosage of GC ≥ 60 mg/day corresponds to a positive predictive value (PPV) of 74.39% and a negative predictive value (NPV) of 51.47%.

## 4. Discussion

The 18F-FDG-PET/CT scan detects glucose uptake from high glycolytic activity on inflammatory cells in inflamed arteries, resulting in a pronounced enhancement of vessel walls [18,19]. Conducting imaging tests early in the diagnostic process tends to yield more positive results. This outcome is most likely to be closely associated with the administration of GC. Consequently, a higher cumulative dose of GC often leads to a higher proportion of negative results in such imaging tests.

This study was conducted to evaluate the efficacy of the 18F-FDG-PET/CT scan in detecting LVV among patients diagnosed with GCA. The results revealed that patients with LVV who tested positive using the 18F-FDG-PET/CT scan were typically younger compared to those with a negative scan. Notably, there was a decreased likelihood of a positive test result for LVV as the duration from the initial clinical suspicion increased and as higher cumulative doses of GC were administered before performing the scan. Interestingly, patients exhibiting a positive 18F-FDG-PET/CT scan demonstrated lower incidences of HT, DM, and CVD.

Regarding age, our results are consistent with the previous literature, which shows that patients with ecGCA are younger than patients with cGCA [20,21]. Our regression model shows that for each one-year increase in age, the OR of having a positive 18F-FDG-PET/CT scan decreases by 5.1% (OR 0.949 [95% CI: 0.914–0.986]). The relevance of this finding requires future research to understand whether GCA has a different phenotype in younger and older people or if this finding corresponds to a bias in the diagnostic approach and treatment. Prieto-Peña et al. reported that GCA patients with LVV involvement tend to have a mean age below 70 years, whereas older patients tend to have the classic cGCA phenotype [21]. In this line, a recent publication by Hay et al. suggests that in elderly and frail patients suspected of having GCA, initiating treatment with GC at an early stage is a common practice [22].

Concerning the time until the acquisition of the 18F-FDG-PET/CT scan, if compared with performing it within the first three days after the clinical suspicion, our results show that for the different periods of 4–10 days, 11–100 days, or more than 101 days, the likelihood of a positive result was 81%, 74%, and 66%, respectively. Recently, Narváez et al. published a small retrospective series of patients with newly diagnosed GCA receiving high-dose oral GC [23], where the rates of 18F-FDG-PET/CT scan positivity were 54.5% for those treated for less than two weeks, 38.5% for those treated for 2 to 4 weeks, and 25% for those treated for 4 to 6 weeks. We attribute the variance in results mainly to the fact that their study population received high-dose intravenous GC (between 125 mg and 1000 mg methylprednisolone/day for 3–5 days.

In our study, based on data from daily clinical practice, the AUC value was 0.74, with a Youden index > 60 mg/day, which is interpreted as reasonably good. These findings suggest that patients with a cumulative GC dosage above this threshold have a 74.4% probability of a positive PET scan result, while those below this threshold have a 51.5% likelihood of a negative outcome. This dosage threshold could potentially serve as a critical marker for clinical decision making, highlighting its relevance in predicting the effectiveness of GC treatment in influencing PET scan results. This information should be considered in discussions about optimizing GC therapy in clinical practice.

Similar data were reported by Clifford et al. [24], with an AUC of 0.75, and by Prieto-González et al. [25], who reported an AUC of 0.830 for the 18F-FDG-PET/CT scan. Both studies had different and more optimum conditions than ours, being prospective case-controls, with GC treatment periods of less than 3 days [25] and a mean of 11.9 days [24] of prednisone until the patient underwent a 18F-FDG-PET/CT scan.

Finally, although it was not the primary focus of this study, it is relevant to acknowledge the adoption of the 2022 ACR/EULAR criteria, which signify a substantial advancement in the diagnostic framework for GCA. In the cohort analyzed from our study, 261 patients were diagnosed with GCA under the 1990 ACR criteria, whereas a notably higher count of 322 patients satisfied the 2022 ACR/EULAR criteria. This distinction underscores the increased sensitivity and specificity afforded by the revised criteria, which effectively integrate contemporary imaging findings. The 2022 ACR/EULAR criteria have been meticulously designed to encompass a broader spectrum of diagnostic features, thereby aligning more closely with advancements in imaging technology. This alignment enhances the accuracy of GCA diagnoses, facilitating timely and precise therapeutic interventions.

We believe that the clinical significance of the 18F-FDG-PET/CT scan is noteworthy, despite the potential negative impact of the elapsed time or high-dose GC treatment on its diagnostic yield. Indeed, as days go by, and the cumulative GC dose is higher, the vascular wall uptake of 18F-FDG is reduced, which is why an 18F-FDG-PET/CT scan should be performed as soon as possible, mostly within 15 days of the suspicion of a diagnosis. In addition, an 18F-FDG-PET/CT scan provides crucial information about the extent of the disease [12,26]. However, its role as a prognostic factor, particularly in terms of assessing the likelihood of relapse and elucidating its role as a potential biomarker is still unanswered [27,28,29].

Another interesting point in the management of GCA/LVV is related to comorbidities. In our study, we collected data for the presence of HT, DM, DLP, and CVD (Table 1). Only the CVD and DM seemed to have a negative association with a positive 18F-FDG-PET/CT scan (Table 2). The discrepancies between Table 1 and Table 2 can be explained by the different approaches and considerations of each statistical method assessed. The bivariate analysis shows HT, DM, and CVD as having a significant difference in the outcome of the 18F-FDG-PET/CT scan in those patients with GCA. In contrast, in the regression model for patients with a positive 18F-FDG-PET/CT scan, adjusting for other factors considered as potential confounding and interactions for confusing variables, only the DM and CVD decreased the probability of a positive result. While there is substantial knowledge about the increased CVD risk in patients with GCA [30] and the need for therapeutic alternatives in patients with worse prognostic factors or high comorbidity [31], no international publications have addressed the impact of these comorbidities on the 18F-FDG-PET/CT scan results in GCA patients. However, given that atherosclerotic plaque in the wall of large arteries may show significant FDG uptake, an effort should be made to develop a standardized scoring system to differentiate LVV from atherosclerosis. It seems that FDG uptake distribution and the presence of calcifications might help to accurately discriminate atherosclerosis from LVV in this pool of patients [32]. More data are needed to evaluate whether the cutoff points for a positive PET-CT scan should be adjusted in the presence of certain cardiovascular risk factors, which could be mistaken for false positive or negative, as was accomplished with ultrasound cutoff points [33].

We consider limitations worth discussing. As it was based on daily clinical practice, the use of an 18F-FDG-PET/CT scan is restricted, since there is not a protocoled standard diagnostic technique in all participant centers, as well as its limited availability. These facts may have a bias toward underestimating the true prevalence of LVV in these patients. Furthermore, it is important to consider the variability in how data could be collected for 18F-FDG-PET/CT scan results, either by a visual semiquantitative approach or by quantitative criteria with the maximal standardized uptake value (SUVm); however, they were recorded in the same concise form, as either positive or negative, which is crucial for a diagnostic and therapeutic approach.

Another limitation of our study is the lack of a standardized definition for main clinical phenotypes. We only considered cranial and extracranial phenotypes based on the presence or absence of cranial symptoms, respectively. Additionally, an overlap of these phenotypes may exist, (mixed [mGCA]), which was not distinctly categorized due to our dichotomous classification system. Nevertheless, it is plausible to interpret the results from patients with cranial phenotypes who demonstrated LVV on PET-CT scans as indicative of mGCA [34]. Other clinical phenotypes, such as silent-GCA (sGCA), whose predominant complaint could be isolated fever and constitutional syndrome [35], were not possible to interpret from our results.

Future research should focus on developing more fitting imaging protocols for the interpretation of 18F-FDG-PET-CT scans in different scenarios, depending on age and comorbidities, especially those related to atherosclerosis, GC dose, or days elapsed between the clinical suspicion and the test. Additionally, prospective studies to validate and standardize clinical phenotypes, including cGCA, ecGCA, mGCA, and sGCA, could significantly enhance the diagnostic accuracy and patient management strategies in the context of GCA.

## 5. Conclusions

Younger patients have a higher probability of presenting an LVV detected by 18F-FDG-PET/CT. The most frequent affected location in LVV was the thoracic aorta. CVD and DM may play a role in reducing the probability of a PET/CT positive for LVV. Our findings support that the timing of the 18F-FDG-PET/CT examination has an impact on the likelihood of a positive result, with earlier tests having a higher probability of detecting vascular inflammation. Simultaneously, the cumulative dosage of GC is a moderately effective predictor of the outcomes related to PET/CT in these patients.

## Figures and Tables

**Table 1:** General clinical features of GCA patients in whom 18F-FDG-PET/CT scan was performed.

	Total N = 377	Negative 18F-FDG-PET/CT scan N = 132	Positive 18F-FDG-PET/CT scan N = 245	*p*-Value *
**Demographic data**				
Women, n (%)	269 (71.4)	93 (70.5)	176 (71.8)	0.78
Age, years, mean (SD)	73.4 (9.0)	76.2 (8.1)	71.9 (9.0)	0
**Diagnosis, n (%)**				
ACR/EULAR 1990	261 (69.23%)	115 (87.12%)	146 (59.59%)	<0.001
ACR/EULAR 2022	322 (85.41%)	124 (93.94%)	198 (80.82%)	0.001
Objective evidence for diagnosis:				
Positive temporal artery biopsy	129 (50.99%)	56 (50.91%)	73 (51.05%)	0.982
Positive temporal artery ultrasound	88 (52.38%)	45 (58.44%)	43 (47.25%)	0.148
Positive large vessel ultrasound	21 (32.31%)	9 (21.95%)	12 (50.00%)	0.020
Positive axillary ultrasound	11 (27.50%)	5 (20.83%)	6 (37.50%)	0.247
Positive subclavian artery ultrasound	7 (21.21%)	0 (0.00%)	7 (21.21%)	0.001
Positive CT-A/MRI-A	24 (30.38%)	5 (13.16%)	19 (46.34%)	0.001
Clinical criteria	6 (1.59%)	6 (4.55%)	0 (0.00%)	0.001
**Laboratory parameters, mean (SD)**				
ESR, mm/h	77.1 (34.9)	76.3 (30.9)	77.5 (36.9)	0.764
C-reactive protein, mg/L, median (Q1–Q3)	63 (22.4–130)	53.4 (20.3–126)	69 (25.2–134.7)	0.23
Hemoglobin, g/dL	11.6 (1.7)	11.8 (1.6)	11.4 (1.7)	0.032
Platelets, ×10^9^/L	330.2 (271.9)	306.4 (140.3)	343.6 (323.3)	0.22
**Comorbidities ^ 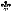 ^, n (%)**				
Hypertension	229 (61.9)	92 (70.8)	137 (57.1)	0.01
Diabetes mellitus	71 (19.4)	33 (25.8)	38 (16.0)	0.024
Dyslipidemia	186 (50.5)	71 (54.6)	115 (48.3)	0.248
Cardiovascular disease	70 (20)	40 (31.5)	30 (13.5)	<0.001
Smoking	41 (32.3)	7 (17.5)	34 (39.1)	0.016
**Clinical phenotypes, n (%)**				
Cranial	250 (69.83)	103 (82.40)	147 (63.09)	<0.001
Extracranial	108 (30.17)	22 (17.60)	86 (36.91)	
**Clinical manifestations, n (%)**				
***Cranial***				
Recent-onset headache	250 (67.39%)	103 (78.63%)	147 (61.25%)	0.001
Temporal artery tenderness or decreased pulsation	127 (36.18%)	57 (45.97%)	70 (30.84%)	0.005
Hypersensitive scalp	80 (24.24%)	30 (26.32%)	50 (23.15%)	0.523
Facial pain	38 (11.95%)	16 (14.04%)	22 (10.78%)	0.391
Dysphagia	14 (4.78%)	7 (7.00%)	7 (3.63%)	0.199
Jaw claudication	103 (28.93%)	43 (34.40%)	60 (25.97%)	0.094
Visual symptoms	103 (27.99%)	53 (41.41%)	50 (20.83%)	<0.001
Vertigo	31 (10.95%)	13 (13.27%)	18 (9.73%)	0.365
Hearing loss	6 (2.17%)	2 (2.08%)	4 (2.21%)	0.945
Transitory ischemic attack	9 (2.51%)	3 (2.42%)	6 (2.56%)	0.934
Stroke	18 (5.01%)	6 (4.84%)	12 (5.11%)	0.912
***Extracranial***				
Polymyalgia rheumatica	166 (45.6%)	64 (49.61%)	102 (43.40%)	0.255
Claudication-lower limbs	35 (10.23%)	13 (10.92%)	22 (9.87%)	0.758
Claudication-upper limbs	34 (9.97%)	13 (10.83%)	21 (9.50%)	0.695
Peripheral synovitis	31 (9.04%)	14 (11.57%)	17 (7.66%)	0.227
***General***				
Asthenia	240 (67.61%)	69 (56.10%)	171 (73.71%)	0.001
Weight loss	149 (43.44%)	41 (33.61%)	108 (48.87%)	0.006
Fever/low-grade fever	127 (38.37%)	43 (36.75%)	84 (39.25%)	0.655
**Delay from the clinical suspicion to the 18F-FDG-PET/CT scan, n (%)**				
0–3 days	188 (53.3)	29 (26.9)	159 (64.9)	<0.001
4–10 days	41 (11.6)	16 (14.8)	25 (10.2)	
11–100 days	77 (21.8)	39 (36.1)	38 (15.5)	
More than 101 days	47 (13.3)	24 (22.2)	23 (9.4)	
**Glucocorticoids treatment before the 18F-FDG-PET/CT scan, n (%)**				
Glucocorticoids (oral or IV)	259 (68.7)	113 (85.6)	146 (59.6)	<0.001
No glucocorticoids	118 (31.3)	19 (14.4)	99 (40.4)	
**Outcomes, n (%)**				
Exitus	19 (5.04%)	10 (7.58%)	9 (3.67%)	0.099
Relapses	74 (19.63%)	31 (23.48%)	43 (17.55%)	0.166
Remission	33 (10.65%)	7 (6.60%)	26 (12.75%)	0.096
Serious adverse events	21 (5.57%)	14 (10.61%)	7 (2.86%)	0.002

Abbreviations: GCA: giant cell arteritis, CT-A: computed tomography angiography, MRI-A: magnetic resonance imaging angiography, ESR: erythrocyte sedimentation rate. ^
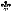
^ n for patients with hypertension was 370, diabetes mellitus 366, dyslipidemia 368, cardiovascular disease 350, and smoking 127. Data are obtained through bivariate analysis. * *p*-value refers to the statistical difference between patients with the listed characteristics and those without. In bold, *p* values < 0.05.

**Table 2:** Regression model for patients with GCA and positive 18F-FDG-PET/TC.

Variables	OR [95% CI]
Age	0.949 ** [0.914–0.986]
Gender	0.610 [0.316–1.179]
Hypertension	1.065 [0.550–2.064]
Diabetes mellitus	0.482 * [0.238–0.978]
Dyslipidemia	0.926 [0.512–1.1675]
Cardiovascular disease	0.439 * [0.211–0.914]
Cranial GCA	1.206 [0.333–4.371]
Extracranial GCA	1.854 [0.455–7.554]
Oral glucocorticoids	0.984 [0.661–1.467]
Intravenous glucocorticoids	0.559 [0.222–1.409]
Days until the 18F-FDG-PET/TC was performed (ref. group 0–3 days)	
4–10 days	0.335 * [0.143–0.783]
11–100 days	0.255 ** [0.125–0.523]
More than 101 days	0.189 ** [0.610–0.587]

Abbreviations: GCA: giant cell arteritis. * *p* < 0.05, ** *p* < 0.01. In bold, significant confidence intervals.

## Data Availability

The original contributions presented in the study are included in the article, further inquiries can be directed to the corresponding authors.
